# THAP9-AS1 Promotes Tumorigenesis and Reduces ROS Generation through the JAK2/STAT3 Signaling Pathway by Increasing SOCS3 Promoter Methylation in Osteosarcoma

**DOI:** 10.1155/2021/5620475

**Published:** 2021-10-14

**Authors:** Shuai Yang, Bing Wang, Chang Liu, Qizun Wang, Ronghuan Wang, Weiliang Su, Youfu Zhu, Maohua Li, Zhaoyang Guo, Xiaolin Wu, Feng Chen

**Affiliations:** ^1^Department of Orthopedics, The Affiliated Hospital of Qingdao University, Qingdao, China 266003; ^2^Department of Orthopedics, Dezhou Municipal Hospital, Dezhou, China 253000

## Abstract

Increasing studies have demonstrated that dysfunction of long noncoding RNAs (lncRNAs) plays critical roles in the development of human cancers. THAP9-AS1 has been reported to be dysregulated and associated with tumor progression in some cancers. However, the function and mechanism of THAP9-AS1 in osteosarcoma (OS) remain unclear. In the present study, we found that the expression of THAP9-AS1 was significantly upregulated in OS tissues and associated with the advanced stage of tumors and poor prognosis of patients. Blast comparison results showed that the SOCS3 promoter region and THAP9-AS1 had base complementary pairing binding sites. The interactions between THAP9-AS1, DNA methyltransferases (DNMTs), and SOCS3 were assessed by RIP and ChIP assays. The results of methylation-specific PCR (MSP) and bisulfite sequencing PCR (BSP) validated that THAP9-AS1 enhanced the methylation level of the SOCS3 promoter. The mRNA levels of SOCS3 in OS cells could be reversed by the demethylation agent 5-aza-2′-deoxycytidine. The mRNA expression of SOCS3 was downregulated in OS tissues and negatively correlated with THAP9-AS1 expression in tumors. Moreover, the western blot and immunofluorescence (IF) assay data showed that THAP9-AS1 activated the JAK2/STAT3 signaling pathway by upregulating p-JAK2 and p-STAT3 and the nuclear translocation of p-STAT3. Functionally, ectopic expression of THAP9-AS1 promoted cell proliferation, migration, and invasion and inhibited apoptosis, and this phenomenon could be reversed by SOCS3. Introduction of the JAK/STAT inhibitor AG490 partially abolished the stimulative effect of THAP9-AS1 on cellular processes. In addition, THAP9-AS1 decreased oxidative stress by reducing reactive oxygen species (ROS) and enhancing the mitochondrial membrane potential of OS cells via the SOCS3/JAK2/STAT3 pathway. Stable overexpression of THAP9-AS1 contributed to tumor growth and metastasis in vivo. In total, our findings suggested that upregulation of THAP9-AS1 might recruit DNMTs to epigenetically inhibit SOCS3, thereby activating the JAK2/STAT3 signaling pathway and oncogenesis of OS. These results provide novel insights for the understanding of OS progression.

## 1. Introduction

Osteosarcoma (OS) is one of the most common malignant bone tumors and occurs more frequently in long bones in children and adolescents [1]. Lung metastasis is often observed in many OS patients upon preliminary diagnosis, and the 5-year survival rate of these patients remains less than 20% [2]. Therefore, a greater depth of insight into the molecular mechanisms involved in OS is essential and useful for identifying new biomarkers and novel treatment strategies.

Long noncoding RNAs (lncRNAs), a group of noncoding RNAs, are longer than 200 nucleotides in length and lack protein coding capacity [3]. Dysregulation of lncRNAs is related to a wide range of tumor cellular processes, such as proliferation, apoptosis, invasion, autophagy, and angiogenesis [4]. Several lncRNAs were identified as potential diagnostic and prognostic markers of OS patients and acted as oncogenes or tumor suppressors in the development of OS. For example, TTN-AS1 increased cell growth and drug resistance by regulating miR-134–5p/MBTD1 [5]. KCNQ1OT1 acts as a competing endogenous RNA (ceRNA) to regulate ALDOA by sponging miR-34c and promotes the Warburg effect in OS cells [6]. Our previous study revealed that PVT1 contributed to glucose metabolism, cell proliferation, and motility through the miR-497/HK2 pathway in OS cells [7]. Another mechanism by which lncRNAs regulate gene expression and tumor progression is by regulating promoter DNA methylation through recruiting DNA methyltransferases (DNMTs) [8, 9]. THAP9-AS1, a long noncoding RNA that is located on 4q21.22, was previously discovered to be upregulated in breast cancer by using next-generation deep sequencing [10]. THAP9-AS1 expression was positively correlated with advanced tumor stages and poor prognosis; knockdown of THAP9-AS1 suppressed cell proliferation and invasion and enhanced apoptosis in esophageal squamous cell carcinoma (ESCC) [11]. THAP9-AS1 was identified as a promotor of pancreatic ductal adenocarcinoma (PDAC) progression by activating YAP signaling, which in turn also modulates THAP9-AS1 transcription [12]. However, the function and mechanism of THAP9-AS1 in the development of OS remain unknown.

Aberrant JAK/STAT signaling has been demonstrated to contribute to cancer progression and metastatic development in various cancers. Activation of the JAK2/STAT3 signaling pathway is involved in LCP1-induced cell proliferation, migration, and invasion in OS [13]. Overexpression of PARK2 inhibited tumor growth, invasion, and angiogenesis, probably through suppressing the JAK2/STAT3/VEGF signaling pathway in OS [14]. Targeting the JAK2/STAT3 pathway is currently one of the most promising therapeutic strategies in the treatment of OS [15, 16]. Suppressors of cytokine signaling (SOCS) proteins can attenuate JAK/STAT signaling by interfering with the activity of JAK kinases [17]. However, the cross-talk between lncRNAs and the JAK2/STAT3 signaling pathway in OS progression remains largely unknown.

This study is aimed at investigating the biological function of THAP9-AS1 in OS and the underlying mechanisms. Our results mainly revealed that THAP9-AS1 was upregulated in OS and negatively correlated with the prognosis of patients. Overexpression of THAP9-AS1 promoted cell proliferation and metastasis in vitro and in vivo and reduced oxidative stress, likely by regulating SOCS3 methylation and expression and the downstream JAK2/STAT3 signaling pathway.

## 2. Materials and Methods

### 2.1. Clinical Samples and Cell Lines

Forty pairs of OS tissues and their matched adjacent nontumor tissues (at least 3 cm away from the tumor) were collected from patients who underwent surgery at the Affiliated Hospital of Qingdao University between March 2015 and July 2016. This study was approved by the Research Ethics Committee of the Affiliated Hospital of Qingdao University. All tissue specimens were recognised by pathologists. Informed consents were obtained from patients. None of the patients received chemotherapy before surgery. Tissue samples were collected during surgery and stored at -80°C until use. The clinicopathological features of the patients were recorded and are summarized in Table 1.

Four human OS cell lines (143B, MG63, SaOS-2, and U2OS) and a normal human osteoblast cell line (hFOB1.19) were obtained from the China Academy of Sciences (Shanghai, China) and maintained in a humidified incubator at 37°C with a 5% CO_2_ atmosphere.

### 2.2. RNA Extraction and Quantitative Real-Time PCR (RT–PCR)

Total RNA from cells and tissue samples was isolated by TRIzol reagent (Invitrogen, Grand Island, NY, USA), and RNA concentration and purity were measured using a NanoDrop 2000 (Thermo, Waltham, MA, USA). A reverse transcription kit (Takara, Japan) was used to generate cDNA from 2 *μ*g of RNA from each sample. Quantitative PCR was performed using SYBR Green Master Mix (Thermo) on a LightCycler 480 System (Roche, Basel, Switzerland). The specific primer sequences were as follows: THAP9-AS1, 5′-TTGCACCCAGATCAGCTACA-3′ (forward) and 5′-GTGTTTTCGGTGACTGTCCC-3′ (reverse); SOCS3, 5′-AGAGAGAGGTCCTGAGGG-3′ (forward), 5′-ATCACACACGTTGCAGAAC-3′ (reverse). GAPDH was used as an endogenous control. The relative expression level was calculated using the 2^-*ΔΔ*Ct^ method, and the experiment was performed in triplicate.

### 2.3. RNAi and Transfection

For RNA interference, small interfering RNAs (siRNAs) that targeted THAP9-AS1 (si-THAP9-AS1, sequence: 5′-GAAGAGCCTATTACATCTACT-3′), DNMT1 (si-DNMT1, sequence: 5′-GGAAAGAGACAGCTTAACAGAAA-3′), DNMT3a (si-DNMT3a, sequence: 5′-GCTGAGAAGAAAGCCAAGGTCAT-3′), and DNMT3b (si-DNMT3b, sequence: 5′-GGGAAACCAGGACTCGTTCAGAAA-3′) were designed. All siRNAs and control siRNA (si-ctrl: 5′-GCGCACTGATCTCGATATCTGT-3′) were purchased from GenePharma (Shanghai, China). Full-length human THP9-AS1 (ENST00000504520) and SOCS3 (NM_003955) cDNA expression plasmids (THAP9-AS1 and pcSOCS3) and the empty control vector (vector) were purchased from Sangon Biotech (China). OS cells were transfected using Lipofectamine 3000 reagent (Invitrogen) according to the manufacturer's instructions. To generate clones stably overexpressing THAP9-AS1, MG63 cells were infected at an MOI of 50 with a lentiviral vector encoding the THAP9-AS1 gene sequence or an empty vector control. Stable clones were selected for 2 weeks using puromycin.

### 2.4. Subcellular Fractionation Assay

Nuclear and cytoplasmic RNA was isolated using a PARIS™ kit (Thermo). After purification and DNase I treatment, RNA from the isolated nuclear and cytoplasmic fractions was reverse transcribed and used for PCR to detect THAP9-AS1. U6 and GAPDH were used as endogenous controls for the nucleus and cytoplasm, respectively.

### 2.5. Fluorescence In Situ Hybridization (FISH)

Cy3-labeled FISH probes were designed and synthesized by GenePharma (Shanghai, China). Cells were fixed with ice-cold 4% paraformaldehyde for 20 min and blocked with prehybridization buffer for 30 min. Then, cells were incubated with THAP9-AS1 FISH probe in hybridization buffer in the dark at 37°C overnight, washed three times, incubated with DAPI for 15 min, and then observed using a confocal fluorescence microscope (Olympus, Japan).

### 2.6. Luciferase Activity Assay

*Wild-type* SOCS3 promoter (pSOCS3-wt) containing THAP9-AS1 binding sites and SOCS3 mutant type (pSOCS3-mut) were ligated into pGLO vectors. Either pGLO-pSOCS3-wt or pGLO-pSOCS3-mut was cotransfected with THAP9-AS1 or control vector into MG63 cells. After 24 h of transfection, the cells were collected, and luciferase activity was detected using a Dual-Luciferase Reporter Assay System (Promega, Madison, WI, USA).

### 2.7. RNA Immunoprecipitation (RIP)

A Magna RIP RNA-Binding Protein Immunoprecipitation kit (Millipore, Billerica, MA, USA) was used in this study. Briefly, MG63 cells were treated with lysis buffer containing protease inhibitor and ribonuclease inhibitor. After centrifugation, the supernatant was collected and incubated with 1 *μ*g of specific antibodies, including anti-IgG (ab109489; Abcam, Cambridge, UK), DNMT1 antibody (ab13537), DNMT3a antibody (ab2850), and DNMT3b antibody (ab2851). After incubation with proteins using A/G-beads (Thermo) at 4°C overnight, the RNA was isolated for subsequent qRT–PCR assay.

### 2.8. Chromatin Immunoprecipitation (ChIP)

The enrichment of DNMT1, DNMT3a, and DNMT3b in the SOCS3 promoter region was assessed using a ChIP kit (Millipore). MG63 cells were incubated until cell confluence reached 70–80% and then fixed in 1% formaldehyde for 10 min to cross-link DNA and protein. Cells were randomly lysed using ultrasonic treatment to obtain 200–1,000 bp DNA fragments. After centrifugation at 4°C, the supernatant was collected and incubated with the following specific antibodies: anti-DNMT1, anti-DNMT3a, or anti-DNMT3b. Mouse IgG was used as the negative control. After decrosslinking at 65°C overnight, DNA was extracted, purified, and collected for PCR assay.

### 2.9. Demethylation Treatment, Methylation-Specific PCR (MSP), and Bisulfite Sequencing PCR (BSP) Assays

For the demethylation assay, different concentrations (0, 2.5 *μ*M, 5 *μ*M, and 10 *μ*M) of 5-aza-2′-deoxycytidine (5-aza; Sigma, St Louis, MO, USA) were used to treat OS cells for three days. Fresh medium containing 5-aza was replaced every 24 hs. After RNA isolation, the mRNA levels of SOCS3 in different groups were determined by PCR assay. For the MSP assay, genomic DNA from cells was extracted using the Genomic DNA Purification Kit (Qiagen, Hilden, Germany) and bisulfite modified using the EpiTect Fast DNA Bisulfite Kit (Qiagen). SOCS3 promoter methylation-specific primers were as follows: methylated, 5′-TGTTTTTGTTCGCGGTTC-3′ (forward) and 5′-CCCCGATTCCTAAAACTACG-3′ (reverse); unmethylated, 5′-TTTTGTTTTTGTTTGTGGTTT-3′ (forward), and 5′-CCCCAATTCCTAAAACTACA-3′ (reverse). The PCR product was 128 bp and then visualized in a 2% agarose gel. For the BSP assay, the promoter region of SOCS3 (-711 to -333 bp relative to the transcription start site, including 27 CpG sites) was analyzed. Primer sequences were as follows: 5′-GGGTTGGTAAAGAATTTGGTAGT-3′ (forward) and 5′-CCCCTCCCTTCTAAAAAAACTA-3′ (reverse). The results of the BSP assay are presented in lollipop diagrams.

### 2.10. Cell Proliferation Assay

MTT method was used to evaluate cell proliferation. In brief, cells were seeded in 96-well plates (1 × 10^4^ cells/well) and incubated at 37°C. At the indicated time points (0, 24, 48, and 72 h), the medium was removed, and MTT (20 *μ*L; 5 mg/mL) was added to each well. After incubation for another 4 hours, DMSO was added to each well, and the absorbance was measured at 490 nm by a microplate reader (Bio-Tek, Germany).

### 2.11. Cell Apoptosis Assay

A total of 2 × 10^5^ cells were cultured in 6-well plates. After being harvested and resuspended in 100 *μ*L of Annexin-V-binding buffer, the cells were incubated with Annexin-V-FITC and propidium iodide (PI, 1 *μ*g/mL). After incubation for 15 minutes in the dark, the stained cells were measured using a BD FACSCalibur (BD Bioscience, San Diego, CA, USA), and the analysis was performed through flow cytometry (BD Bioscience).

### 2.12. Cell Migration and Invasion Assays

Transwell chambers were used to measure cell migration and invasion. The upper surface of the filter (8.0 *μ*m; Biosciences, Heidelberg, Germany) with and without Matrigel coating (BD Biosciences) was used in invasion and migration assays, respectively. A total of 3 × 10^4^ posttransfection OS cells in 100 *μ*L serum-free DMEM were added to the upper chambers, and the lower chambers were filled with 700 *μ*L DMEM with 20% FBS. After incubation for 24 h, the membranes were fixed with 4% methanol and stained with 0.1% crystal violet. The stained cells were counted in five random fields.

### 2.13. Analysis of Reactive Oxygen Species (ROS) and Mitochondrial Membrane Potential

For detection of ROS generation, OS cells were seeded in a 24-well plate (1 × 10^5^ cells/well). After being washed with PBS, the medium was replaced with 500 *μ*L DCF-DA and incubated at 37°C for 20 min. After washing, the cells were observed under a fluorescence microscope. The mitochondrial membrane potential of cells was evaluated using the JC-1 probe staining method (Beyotime, China). In brief, the treated cells were seeded onto 6-well plates and cultured for 24 h. After washing with PBS and adding complete medium, the cells were immersed in JC-1 working solution and incubated at 37°C for 20 min. After the cells were washed with cold JC-1 staining buffer three times, the MMP of the cells was detected by fluorescence microscopy (Leica, Germany). Finally, the green/red fluorescence intensity ratio can indirectly reflect the state of the mitochondrion.

### 2.14. Immunofluorescence (IF) Staining

The transfected cells were fixed with 4% paraformaldehyde, washed three times with PBS, and permeabilized with 0.2% Triton X-100/PBS solution. Then, the cells were incubated with an anti-p-STAT3 antibody (ab76315) overnight at 4°C, washed with PBS, and incubated with a fluorescence-conjugated secondary antibody. After that, the cells were incubated with 4′,6-diamidine-2′-phenylindole dihydrochloride (DAPI) for 3 min at room temperature. Fluorescent images were obtained using a confocal microscope (Olympus).

### 2.15. Western Blot Analysis

Protein lysates were extracted using RIPA buffer, and 20 *μ*g of protein was separated on 10% polyacrylamide gels and transferred onto PVDF membranes. The membranes were then blocked with 5% nonfat milk in Tris-buffered saline containing 0.05% Tween 20 (TBST) at room temperature for 1 h. The membranes were incubated with antibodies, including anti-SOCS3 (ab16030), anti-phosphorylated JAK2 (anti-p-JAK2, ab32101), anti-phosphorylated STAT3 (anti-p-STAT3, ab76315), anti-STAT3 (ab68153), anti-Bcl-2 (ab182858), anti-E-cadherin (ab1416), anti-N-cadherin (ab18203), anti-*β*-actin (ab8227), and anti-GAPDH (ab9485), overnight at 4°C. After washing with PBST, the bound antibodies were detected by horseradish peroxidase-conjugated secondary antibodies, and the blots were developed using ECL reagents.

### 2.16. Xenograft Tumor Growth and In Vivo Metastasis Assays

Animal experiments were approved by the Institutional Committee for Animal Research of the Affiliated Hospital of Qingdao University. Ten BALB/c nude mice were used in this research (*n* = 5 for each group), and 5 × 10^6^ cells transfected with THAP9-AS1 or vector control cells were subcutaneously injected into the flanks of mice. Tumor volume (width^2^ × length/2) was measured every five days. Finally, mice were sacrificed, and samples were processed for further research. For the in vivo metastasis assay, MG63 cells (1 × 10^7^) expressing THAP9-AS1 or vector control were injected into the tail vein of BALB/c nude mice (*n* = 5/group). All mice were sacrificed after 49 days. The lung tissues were obtained and prepared into paraffin-embedded sections. HE staining was performed to check the metastatic nodules of the lungs under a light microscope. At least five random sections per lung tissue were analyzed.

### 2.17. Immunohistochemical (IHC) Staining

The tumor tissues were paraffin-embedded and cut into 5 *μ*m thick slides for immunohistochemical analysis. After incubating in 0.15% Triton X-100 and blocking with 1% goat serum albumin in modified D-PBS Tween-20, the sections were incubated overnight with Ki-67 and p-STAT3 antibodies. The sections were incubated with secondary antibodies at 37°C for 30 min, and the slides were analyzed under a light microscope.

### 2.18. Statistical Analysis

Data were expressed as the mean ± standard deviation (SD). Statistical analysis was performed using the SPSS 16.0 software (SPSS, Chicago, IL, USA). Kaplan–Meier survival and log-rank tests were used for survival analysis. The correlation between THAP9-AS1 expression and SOCS3 expression in OS tissues was determined using Pearson's correlation analysis. Two-tailed Student's *t*-test was applied to compare the differences between two groups, and one-way analysis of variance (ANOVA) was used to compare the differences among three independent groups. *p* < 0.05 was considered statistically significant.

## 3. Results

### 3.1. THAP9-AS1 Is Upregulated in OS Tissues and Cell Lines

**Figure 1:**
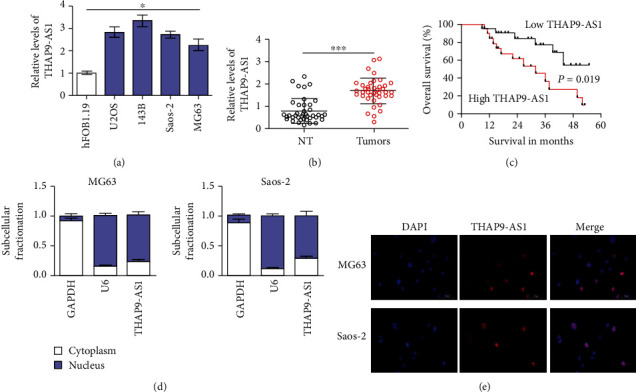
THAP9-AS1 was highly expressed in OS tissues and cells. (a) The expression of THAP9-AS1 was determined in OS cells. (b) The expression levels of THAP9-AS1 were significantly higher in OS tissues than in normal control tissues. (c) Patients with high expression of THAP9-AS1 (>mean) had relatively shorter survival times. (d) The results of the subcellular fractionation assay showed the nuclear expression of THAP9-AS1 in MG63 cells. (e) Subcellular localization (×200) of THAP9-AS1 in MG63 cells was detected by FISH. ^∗^*p* < 0.05; ^∗∗∗^*p* < 0.001; NT: nontumor tissues.

First, qRT–PCR assays were performed to evaluate the expression of THAP9-AS1 in OS cells and tissues. We found that the expression levels of THAP9-AS1 were significantly upregulated in OS cells compared with hFOB1.19 cells (*p* < 0.05; Figure 1(a)). The expression of THAP9-AS1 was dramatically higher in OS tissues than in nontumor tissues (1.67 ± 0.59 vs. 0.78 ± 0.56, *p* < 0.001; Figure 1(b)). To investigate the impact of THAP9-AS1 expression on patient survival, we divided cases into high and low groups according to the mean level of THAP9-AS1 in OS tissues. Patients with a high expression level of THAP9-AS1 tended to have a poor overall survival probability (*p* = 0.019; Figure 1(c)). The location of THAP9-AS1 in OS cells was investigated. As shown in Figures 1(d) and 1(e), the results of subcellular fractionation and FISH assays indicated that THAP9-AS1 might be mainly expressed in the nucleus of OS cells.

### 3.2. THAP9-AS1 Promoted Methylation of the SOCS3 Promoter Region

**Figure 2:**
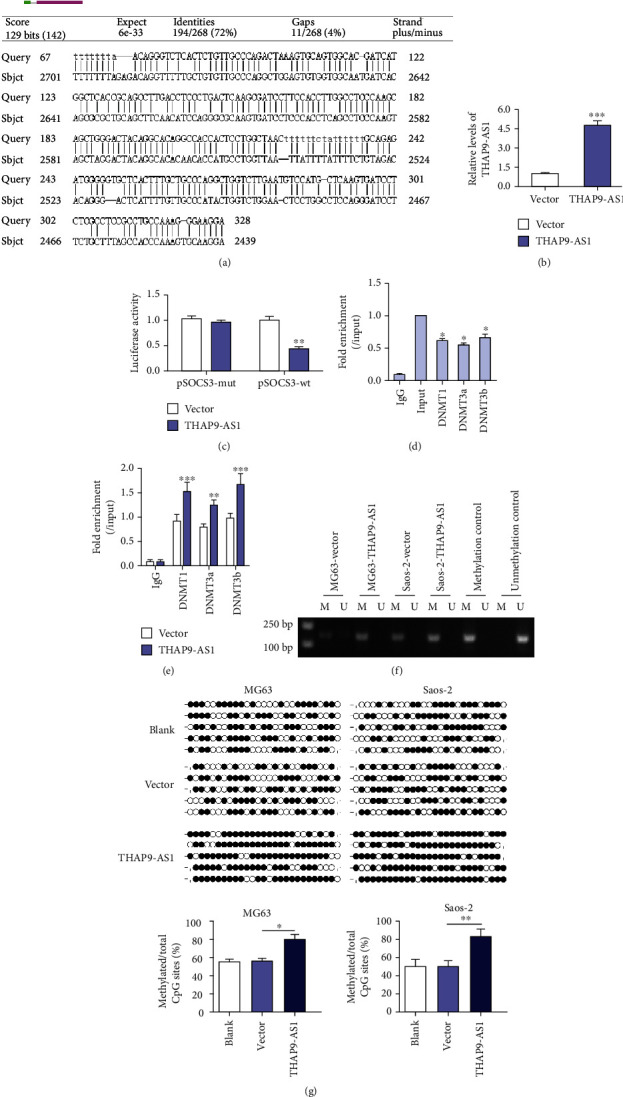
THAP9-AS1 promoted SOCS3 promoter methylation by recruiting DNMTs. (a) Results of BLAST comparison between THAP9-AS1 and the SOCS3 promoter region. (b) The expression of THAP9-AS1 was significantly upregulated in MG63 cells after transfection with THAP9-AS1-expressing vectors. (c) The binding of THAP9-AS1 to the SOCS3 promoter was evaluated by dual-luciferase reporter gene assay. (d) Fold enrichment of DNMT1, DNMT3a, and DNMT3b in the SOCS3 promoter was determined by ChIP assay. (e) RIP was used to detect the binding between THAP9-AS1 and DNMTs. (f) MSP was conducted to verify the methylation status of SOCS3. U: unmethylation; M: methylation. (g) The results of the BSP assay validated that overexpression of THAP9-AS1 induced promoter hypermethylation of SOCS3; black circle, methylated CpG sites; white circle, unmethylated CpG sites. THAP9-AS1- and THAP9-AS1-expressing vectors; vector, empty vector control. pSOCS3-mut: mutants of the SOCS3 promoter; pSOCS3-wt: wild-type SOCS3 promoter. ^∗^*p* < 0.05; ^∗∗^*p* < 0.01; ^∗∗∗^*p* < 0.001.

By applying the RPIseq (http://pridb.gdcb.iastate.edu/RPISeq/) database, THAP9-AS1 was predicted to be combined with DNMT1, DNMT3a, and DNMT3b with high prediction scores (data not shown). Thus, we proposed a hypothesis that THAP9-AS1 may affect gene expression through epigenetic modification. Blast comparison results showed that the promoter regions of SOCS3 and THAP9-AS1 had base complementary pairing binding sites (Figure 2(a)). The expression of THAP9-AS1 was significantly upregulated in MG63 cells after transfection with THAP9-AS1-expressing vectors (*p* < 0.001; Figure 2(b)). We found that compared with the vector control group, the luciferase activity of cells transfected with pSOCS3-wt in the THAP9-AS1 overexpression group was reduced significantly (*p* < 0.01, Figure 2(c)). To investigate the binding of THAP9-AS1 to the SOCS3 promoter region, ChIP and RIP assays were performed in MG63 cells. The results of the ChIP assay showed that the methyltransferases DNMT1, DNMT3a, and DNMT3b were enriched in the promoter of SOCS3 (Figure 2(d)). The results of the RIP assay showed that THAP9-AS1 exhibited a higher conjugation rate with DNMT1, DNMT3a, and DNMT3b (Figure 2(e)). Furthermore, MSP assays validated that overexpression of THAP9-AS1 elevated the methylation of the SOCS3 promoter in both MG63 and Saos-2 cells (Figure 2(f)). Ectopic expression of THAP9-AS1 increased the number of methylated CpG sites in MG63 and Saos-2 cells compared with the vector control group (Figure 2(g)).

### 3.3. THAP9-AS1 Negatively Regulated SOCS3 in OS Cells and Tissues

**Figure 3:**
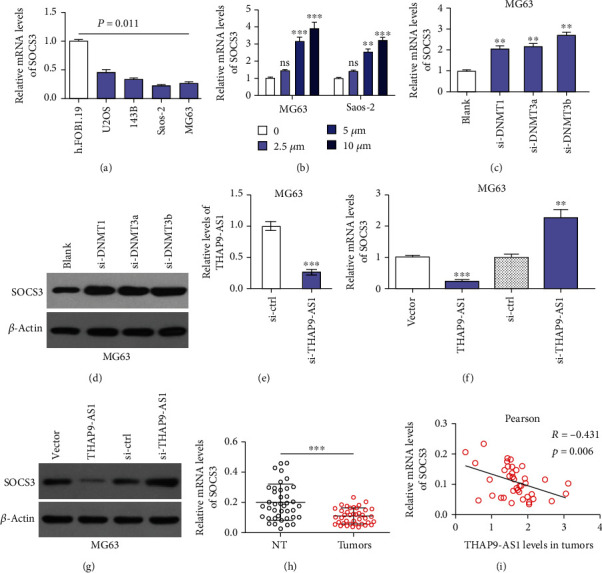
THAP9-AS1 negatively regulated SOCS3 in OS cells and tissues. (a) The mRNA levels of SOCS3 were downregulated in OS cells. (b) The mRNA levels of SOCS3 were restored in OS cells treated with 5-aza. (c, d) Knockdown of DNMTs in MG63 cells increased the mRNA and protein expression of SOCS3. (e) The expression of THAP9-AS1 was effectively downregulated in MG63 cells after transfection with si-THAP9-AS1. (f, g) The mRNA and protein expression of SOCS3 was inhibited by THAP9-AS1 but increased by si-THAP9-AS1. (h) The mRNA levels of SOCS3 were significantly reduced in OS tissues. (i) The mRNA levels of SOCS3 were inversely correlated with THAP9-AS1 in OS tissues. ^∗∗^*p* < 0.01; ^∗∗∗^*p* < 0.001. ns: no significance; si-DNMTs: siRNAs for knocking down DNMTs; si-THAP9-AS1: siRNA specific for silencing THAP9-AS1; si-ctrl: siRNA negative control.

The PCR results validated that the mRNA levels of SOCS3 were downregulated in OS cells compared with normal control cells (*p* = 0.011, Figure 3(a)). The demethylating agent 5-aza restored the mRNA levels of SOCS3 in a dose-dependent manner in both MG63 and Saos-2 cells (Figure 3(b)). Small interfering RNAs were designed to knockdown DNMTs, and the mRNA levels of SOCS3 were significantly recovered in MG63 cells after transfection with si-DNMT1, si-DNMT3a, and si-DNMT3b (Figure 3(c)). The protein expression of SOCS3 was also reversed after DNMT silencing (Figure 3(d)). The expression of THAP9-AS1 was significantly decreased in MG63 cells after transfection with si-THAP9-AS1 (Figure 3(e)). We found that both the mRNA and protein expression of SOCS3 were inhibited by THAP9-AS1 upregulation but promoted by knockdown of THAP9-AS1 in MG63 cells (Figures 3(f) and 3(g)). The mRNA expression of SOCS3 was decreased in OS tissues (0.199 ± 0.117 vs. 0.108 ± 0.052; *p* < 0.001; Figure 3(h)) and negatively correlated with THAP9-AS1 expression in tumors (Pearson *r* = −0.431, *p* = 0.006; Figure 3(i)). Moreover, we found that the downregulation of SOCS3 was significantly associated with the aggressive phenotypes of tumors (*p* < 0.001) and tumor size (*p* = 0.027; Table 1).

### 3.4. THAP9-AS1 Contributed to JAK2/STAT3 Signaling by Inhibiting SOCS3

**Figure 4:**
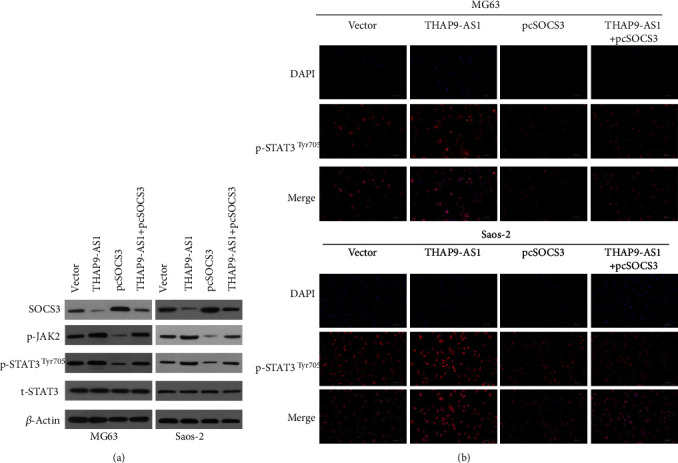
THAP9-AS1 activated the JAK2/STAT3 signaling pathway by regulating SOCS3. (a) THAP9-AS1 induced p-JAK2 and p-STAT3 (Tyr705) protein upregulation, which could be reversed by SOCS3. (b) p-STAT3 nuclear translocation in the different groups of cells was analyzed by IF assay, and representative images are shown; scale bars: 100 *μ*m. pcSOCS3: SOCS3 expressing vectors.

Considering the inhibitory role of SOCS3 in the activation of the JAK2/STAT3 signaling pathway, the effect of THAP9-AS1 on JAK2/STAT3 signaling was investigated. We found that the protein expression of p-JAK2 and p-STAT3 (Tyr705) was induced by THAP9-AS1 but diminished by SOCS3 (Figure 4(a)). Reintroduction of SOCS3 attenuated the stimulatory effect of THAP9-AS1 on p-JAK2 and p-STAT3 (Figure 4(a)). Considering that p-STAT3 in the nucleus is essential for the transcription of its downstream target genes, we examined whether nuclear p-STAT3 accumulation was altered by THAP9-AS1 and SOCS3. The results of the IF assay demonstrated that the nuclear translocation of p-STAT3 protein was induced by THAP9-AS1 but impaired by SOCS3 (Figure 4(b)). These results suggested that STAT3 signaling played a crucial role in THAP9-AS1-regulated cellular function.

### 3.5. The THAP9-AS1/SOCS3/JAK2/STAT3 Signaling Pathway Regulated OS Cellular Function

**Figure 5:**
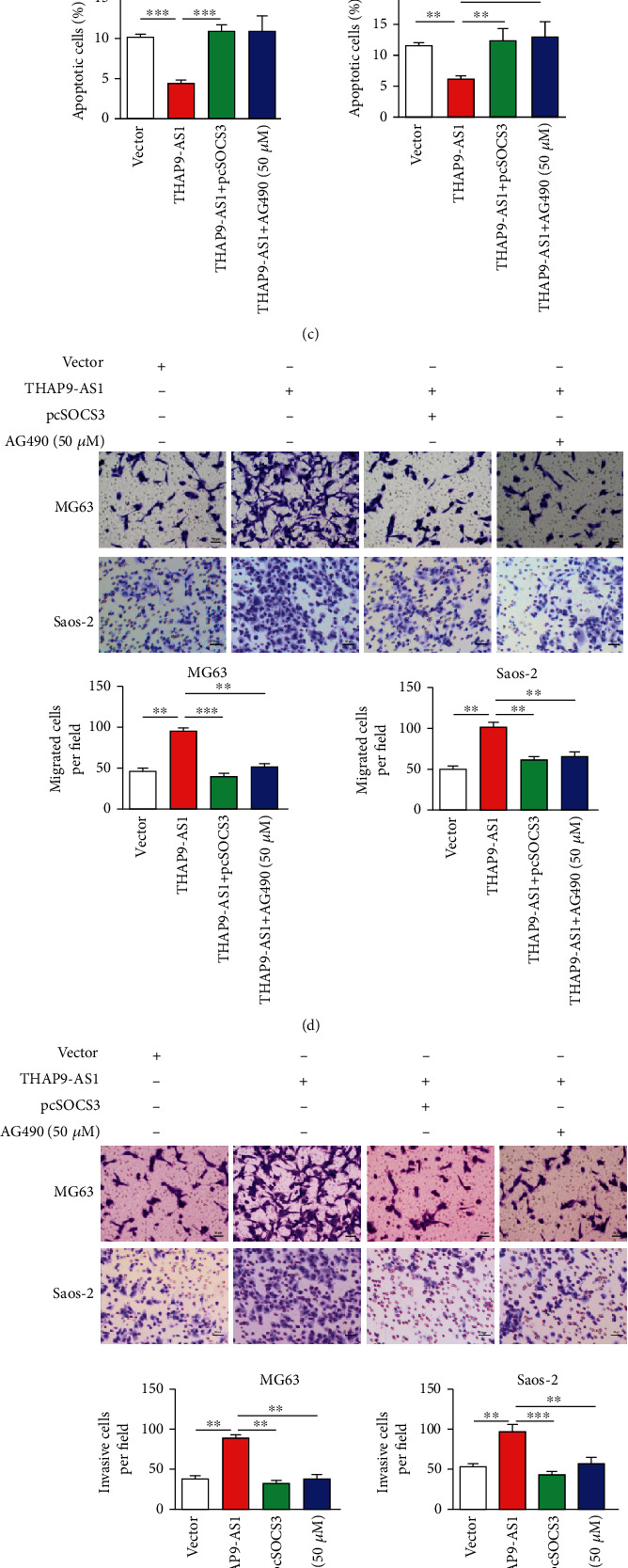
THAP9-AS1 promoted cell growth and invasion in vitro via the SOCS3/JAK2/STAT3 signaling pathway. (a) The protein expression of p-STAT3 was measured. (b) THAP9-AS1 promoted cell proliferation, which could be reversed by either SOCS3 or AG490. (c) Cell apoptosis was inhibited by THAP9-S1 but promoted by either SOCS3 or AG490. (d, e) The number of migrated and invaded cells was increased in the THAP9-AS1 groups and decreased in the SOCS3-expressing and AG490-treated groups; scale bars: 50 *μ*m. (f) THAP9-AS1 promoted EMT progression by decreasing E-cadherin and increasing N-cadherin. ^∗^*p* < 0.05; ^∗∗^*p* < 0.01; ^∗∗∗^*p* < 0.001. AG490: inhibitor of JAK2/STAT3 signaling.

Cells were divided into four groups transfected with different vectors: vector control, THAP9-AS1-expressing vector, THAP9-AS1 plus SOCS3-expressing vector, and THAP9-AS1 plus AG490 (50 *μ*M, JAK2/STAT3 signaling inhibitor). The protein expression of p-STAT3 (Tyr705) was determined to evaluate the transfection efficiency (Figure 5(a)). We found that ectopic expression of THAP9-AS1 promoted cell proliferation, whereas reintroduction of SOCS3 exhibited an inhibitory effect on cell proliferation (Figure 5(b)). Treatment with AG490 abolished the promoting effect of THAP9-AS1 (Figure 5(b)). Cell apoptosis was inhibited by THAP9-AS1 and recovered in the groups treated with either SOCS3 or AG490 (Figure 5(c)). Upregulation of THAP9-AS1 increased the number of migrated and invaded cells, which was impaired by SOCS3 (Figures 5(d) and 5(e)). Inhibition of JAK2/STAT3 signaling by treatment with AG490 significantly reversed the promoting effect of THAP9-AS1 on cell migration and invasion (Figures 5(d) and 5(e)). In addition, the protein expression of Bcl-2 and N-cadherin (a marker of epithelial-mesenchymal transition (EMT)) was elevated, and E-cadherin (a marker of EMT) was suppressed in the THAP9-AS1 groups (Figure 5(f)). These results suggested that THAP9-AS1 contributed to cell growth and that movement might be correlated with the SOCS3/JAK2/STAT3 signaling pathway.

### 3.6. THAP9-AS1 Restrained Oxidative Stress through the SOCS3/JAK2/STAT3 Signaling Pathway

**Figure 6:**
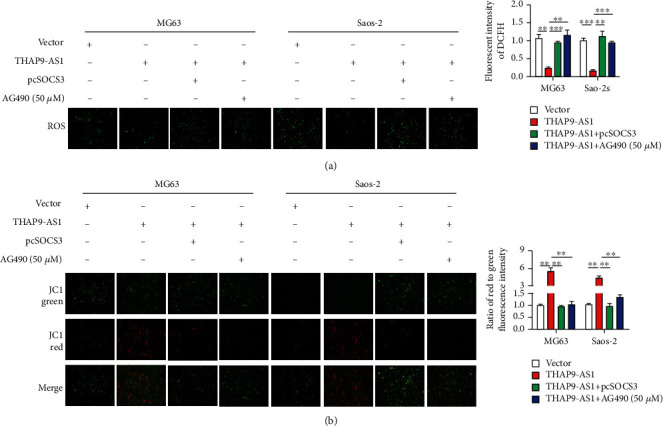
Dysregulation of THAP9-AS1/SOCS3 causes mitochondrial dysfunction. (a) ROS were reduced in the THAP-AS1 groups and restored in the SOCS3 and AG490 treatment groups; scale bars: 100 *μ*m. (b) JC-1 staining was used to observe the mitochondrial membrane potential. The red fluorescence of the JC-1 probe indicates normal mitochondria, and the green fluorescence of the JC-1 probe indicates damaged mitochondrial potential; scale bars: 100 *μ*m. ^∗∗^*p* < 0.01; ^∗∗∗^*p* < 0.001.

We monitored mitochondrial function because the role of mitochondria goes beyond their capacity to create molecular fuel and includes the generation of ROS. As shown in Figure 6(a), compared to the control group, overexpression of THAP9-AS1 notably reduced ROS generation, indicating a decline in cellular oxidative stress. Overexpression of SOCS3 or treatment with AG490 restored ROS levels (Figure 6(a)). The mitochondrial membrane potential was observed using JC-1 staining. As shown in Figure 6(b), the OS cells in the THAP9-AS1 overexpression group exhibited high mitochondrial membrane potential, as evidenced by more red fluorescence. By comparison, the mitochondria in either SOCS3- or AG490-treated cells exhibited more green fluorescence, indicating a reduction in mitochondrial membrane potential. These data indicated that THAP9-AS1 mediated mitochondrial dysfunction in OS cells.

### 3.7. THAP9-AS1 Promoted Tumor Growth and Metastasis In Vivo through JAK2/STAT3 Signaling

**Figure 7:**
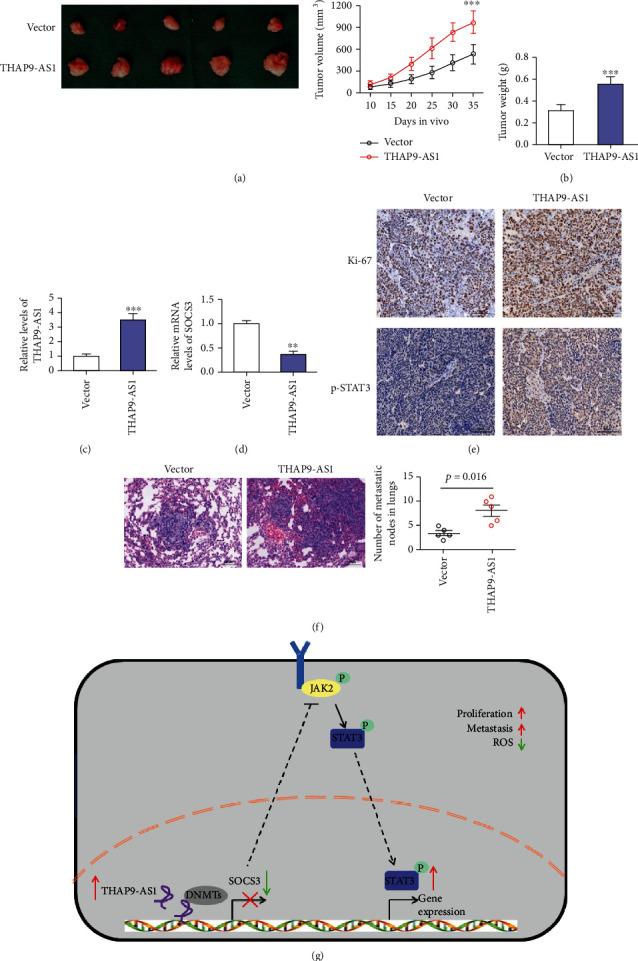
THAP9-AS1 promoted tumor growth and metastasis in vivo. (a, b) Upregulation of THAP9-AS1 increased the volumes and weights of mouse tumors. (c) The expression of THA9-AS1 in mouse tumors was determined. (d) The expression of SOCS3 was decreased in tumors originating from the THAP9-AS1 group. (e) IHC assay for the expression of Ki-67 and p-STAT3 in mouse tumors; scale bars: 100 *μ*m. (f) The number of lung metastatic nodes was increased by THAP9-AS1; HE staining was conducted to verify the lung metastasis foci; scale bars: 100 *μ*m. (g) A schematic showing that THAP9-AS1 contributed to cell proliferation and metastasis and inhibited ROS production, probably through epigenetically suppressing SOCS3, thereby activating the JAK2/STAT3 signaling pathway. ^∗∗^*p* < 0.01; ^∗∗∗^*p* < 0.001.

MG63 cells stably expressing THAP9-AS1 or control vector were injected into mice to evaluate the function of THAP9-AS1 in tumorigenicity. Ectopic expression of THAP9-AS1 led to a relatively larger size of mouse tumors, as well as higher tumor weights (Figures 7(a) and 7(b)). In mouse tumors, THAP9-AS1 was effectively overexpressed in THAP9-AS1-transfected groups (Figure 7(c)). The mRNA expression of SOCS3 was decreased in the THAP9-AS1 groups (Figure 7(d)). The results of the IHC assay validated that Ki-67, a proliferation marker accompanied by p-STAT3, was upregulated in the THAP9-AS1 group (Figure 7(e)). Moreover, we found that the overexpression of THAP9-AS1 contributed to lung metastasis of MG63 cells (Figure 7(f)).

## 4. Discussion

Accumulating evidence has proven that abnormalities in lncRNAs play critical roles in the development of cancers by affecting various signaling pathways. Our findings provide evidence demonstrating that overexpression of THAP9-AS1 leads to promoter hypermethylation of SOCS3, which further silences SOCS3 expression and activates the JAK2/STAT3 signaling pathway, thereby promoting tumorigenesis and metastasis and suppressing oxidative stress.

First, we found that the expression of THAP9-AS1 was increased in OS cells and tissues. High expression of THAP9-AS1 indicated the aggressive phenotypes of tumors and the worse clinical outcome of patients. These data suggested that THAP9-AS1 may play a role in the progression of OS. THAP9-AS1 is overexpressed in PDAC tissues and positively associated with poor survival of patients [12]. High expression levels of THAP9-AS1 were also observed in gastric cancer tissues and could be induced by Helicobacter pylori infection [18]. In ESCC, upregulation of THAP9-AS1 was positively correlated with tumor size, TNM stage, lymph node metastasis, and worse prognosis [11]. According to the microarray expression profile, THAP9-AS1 was upregulated in breast cancer tissues compared with normal control tissues [10]. Thus, dysregulation of THAP9-AS1 may have important roles in a wide range of human malignancies.

The results of subcellular fractionation and FISH assays showed that THAP9-AS1 was mainly expressed in the nucleus of MG63 cells. Thus, THAP9-AS1 may regulate gene expression through an epigenetic mechanism by interacting with DNMTs. Critical DNMT family members, including DNMT1, DNMT3a, and DNMT3b, were closely associated with transcription silencing. The main function of DNMT1 is to maintain the methylation status after DNA synthesis, while DNMT3a and DNMT3b are involved in the de novo methylation of DNA [19]. As an epigenetic modification, DNA methylation is an important cellular process by which methyl groups are added to DNA molecules and leads to the initiation and progression of cancers [20]. High methylation of the SOCS3 promoter has been reported in several cancers, such as lung cancer and hepatocellular and cervical carcinomas [21–23]. The activation of STAT3 signaling and the inhibited expression of SOCS3 may be responsible for aggressive tumor growth in DNMT3b-positive ESCC cells [24]. Previous studies have validated that DNMT1 negatively regulates SOCS3 expression in several types of cancer cells [25–27]. In the present study, we found that the demethylation agent 5-aza restored SOCS3 mRNA expression to different degrees. Silencing of DNMT1, DNMT3a, and DNMT3b restored the mRNA and protein levels of SOCS3 in OS cells. These data suggested that DNA methylation may be involved in the regulation of SOCS3 in OS progression. The results of ChIP, RIP, MSP, and BSP assays validated that THAP9-AS1 might recruit DNMTs to the promoter of SOCS3 and lead to the hypermethylation status of the SOCS3 promoter region. Moreover, the mRNA levels of SOCS3 were significantly reduced in OS tissues and negatively correlated with THAP9-AS1. These data suggested that THAP9-AS1 may be an explanation for the decrease in SOCS3 in the carcinogenesis of OS. Low expression of SOCS3 was associated with TNM stage and tumor size, suggesting that SOCS3 may influence OS.

We found that THAP9-AS1 contributed to the nuclear transfer of p-STAT3 and that the protein expression of p-JAK2 and p-STAT3 (Tyr705) in OS cells was reversed by SOCS3. These data revealed that THAP9-AS1 activated JAK2/STAT3 signaling by inhibiting SOCS3 in OS. MiR-19b, an increased molecule, activates the JAK2/STAT3 signaling pathway by targeting SOCS3 in OS [28]. MiR-4449 promoted cell proliferation and JAK2/STAT3 signaling activity by inhibiting SOCS3 in colorectal cancer [29]. Our data expanded the knowledge of THAP9-AS1 as a possible upstream regulator of the SOCS3/JAK2/STAT3 pathway in OS development.

Functionally, ectopic expression of THAP9-AS1 promoted cell proliferation, migration, and invasion in vitro, which could be reversed by SOCS3. In addition, inhibition of JAK2/STAT3 signaling by introducing AG490 attenuated the cancer-promoting activity of THAP9-AS1. The facilitating role of THAP9-AS1 in tumor growth and lung metastasis was also validated by animal experiments. We suggest that THAP9-AS1 may regulate gene expression and downstream pathways by interacting with DNMTs. However, THAP9-AS1 exerts its oncogenic function by sponging miR-133b and miR-484 in ESCC and PDAC, respectively [11, 12]. It is possible that THAP9-AS1 may affect tumorigenesis through multiple molecular mechanisms in cancers. Furthermore, our data suggested that THAP9-AS1 decreased ROS production and increased the membrane potential of OS cells, suggesting that alteration of oxidative stress may participate in THAP9-AS1-regulated OS progression. It was demonstrated that both nuclear and mitochondrial STAT3 lead to impaired ROS generation and increased ROS scavenging activity [30, 31].

## 5. Conclusions

In summary, our results revealed that THAP9-AS1 was upregulated in OS tissues and correlated with clinical stage. High THAP9-AS1 contributed to the inhibition of SOCS3 and the activation of the JAK2/STAT3 signaling pathway, thus exerting oncogenic functions in OS pathogenesis (Figure 7(g)). We suggest that THAP9-AS1 may serve as a novel biomarker and that THAP9-AS1/SOCS3/JAK2/STAT3 may serve as a therapeutic strategy for the treatment of OS.

## Figures and Tables

**Table 1:** The correlations between clinicopathological features and dysregulation of moleculars.

Characteristics	No. of cases	THAP9-AS1		*p*	SOCS3 mRNA		*p*
	*n* = 40	Low, *n* = 21	High, *n* = 19		Low, *n* = 21	High, *n* = 19	
Sex				0.379			0.707
Female	16	7	9		9	7	
Male	24	14	10		12	12	
Age (years)				0.988			0.528
<20	19	10	9		11	8	
≥20	21	11	10		10	11	
Histological type				0.877			0.398
Osteoblastic	29	15	14		14	15	
Chondroblastic	11	6	5		7	4	
Location				0.841			0.251
Femur	28	15	13		13	15	
Tibia	12	6	6		8	4	
TNM stage				*0.028*			*<0.001*
I, II	22	15	7		6	16	
III	18	6	12		15	3	
Tumor size (cm)				0.355			*0.027*
<5	20	12	8		7	13	
≥5	20	9	11		14	6	

Low: low expression (< mean); high: high expression (≥ mean); *p* < 0.05 are present in italic.

## Data Availability

All data related to this paper may also be requested from the corresponding author.
